# Linking Eco-Energetics and Eco-Hydrology to Select Sites for the Assisted Colonization of Australia’s Rarest Reptile

**DOI:** 10.3390/biology2010001

**Published:** 2012-12-27

**Authors:** Nicola Mitchell, Matthew R. Hipsey, Sophie Arnall, Gavan McGrath, Hasnein Bin Tareque, Gerald Kuchling, Ryan Vogwill, Murugesu Sivapalan, Warren P. Porter, Michael R. Kearney

**Affiliations:** 1 School of Animal Biology, The University of Western Australia, Crawley, Western Australia 6009, Australia; E-Mail: sophie.arnall@uwa.edu.au; 2 School of Earth and Environment, The University of Western Australia, Crawley, Western Australia 6009, Australia; E-Mails: matt.hipsey@uwa.edu.au (M.R.H.); gavan.mcgrath@uwa.edu.au (G.M.); a.m.hasnein@gmail.com (H.B.T.); ryan.vogwill@uwa.edu.au (R.V.); 3 Department of Environment and Conservation, Swan Coastal District, 5 Dundebar Rd., Wanneroo WA 6065, Australia; E-Mail: Gerald.Kuchling@dec.wa.gov.au; 4 Department of Geography, University of Illinois at Urbana-Champaign, Urbana, IL 61801, USA; E-Mail: sivapala@illinois.edu; 5 Department of Zoology, University of Wisconsin, Madison, WI 53706, USA; E-Mail: wpporter@wisc.edu; 6 Department of Zoology, The University of Melbourne, Victoria 3010, Australia; E-Mail: mrke@unimelb.edu.au

**Keywords:** assisted colonization, climate change, rainfall decline, hydroperiod, thermodynamic niche, tortoise, *Pseudemydura umbrina*

## Abstract

Assisted colonization—the deliberate translocation of species from unsuitable to suitable regions—is a controversial management tool that aims to prevent the extinction of populations that are unable to migrate in response to climate change or to survive *in situ*. The identification of suitable translocation sites is therefore a pressing issue. Correlative species distribution models, which are based on occurrence data, are of limited use for site selection for species with historically restricted distributions. In contrast, mechanistic species distribution models hold considerable promise in selecting translocation sites. Here we integrate ecoenergetic and hydrological models to assess the longer-term suitability of the current habitat of one of the world’s rarest chelonians, the Critically Endangered Western Swamp Tortoise (*Psuedemydura umbrina*). Our coupled model allows us to understand the interaction between thermal and hydric constraints on the foraging window of tortoises, based on hydrological projections of its current habitat. The process can then be repeated across a range of future climates to identify regions that would fall within the tortoise’s thermodynamic niche. The predictions indicate that climate change will result in reduced hydroperiods for the tortoises. However, under some climate change scenarios, habitat suitability may remain stable or even improve due to increases in the heat budget. We discuss how our predictions can be integrated with energy budget models that can capture the consequences of these biophysical constraints on growth, reproduction and body condition.

## 1. Introduction

Climate change is occurring at a pace that may prohibit an evolutionary response in some species [1], and species threatened with extinction may need to be translocated to climatically favorable habitats capable of supporting them in the long-term. This process, known variously as assisted colonization, assisted migration or managed relocation [2], has generated much controversy [3,4] but may be the only means of maintaining some species in the wild under future climates or hydrological change [5,6].

A clear candidate for assisted colonization in Australia is the Critically Endangered Western Swamp Tortoise, *Pseudemydura umbrina,* of which less than 50 adults survive in the wild. The species is restricted to two ephemeral swamps on the fringe of Perth, Australia’s fastest growing city [7], where the regional climate has demonstrated a notable shift to drier conditions over the past three decades [8,9] which has reduced the quality of the tortoise’s habitat [10,11]. In particular, the reduction in rainfall and increasing abstraction of groundwater for domestic and agricultural use [12] is shortening the hydroperiod of the swamp habitat, and reducing the recruitment of juveniles into the population [10]. Moreover, long generation times, slow rates of reproduction and low genetic diversity [11] all mean that the tortoise is unlikely to mount an evolutionary response to a changing climate. Human intervention will be necessary to prevent the extinction of this species in the wild—a fact recognized more than 40 years ago and that provided the impetus for a captive breeding program at the Perth Zoo [13] which has now produced more than 500 individuals for release. Translocation protocols are well developed and have demonstrated that captive-bred *P. umbrina* can be successfully introduced into novel habitats, but translocation sites that can offer good habitat under future climates are needed to ensure the persistence of populations in the wild [11]. Given that declining rainfall in southwestern Australia is expected to continue [14], future translocations sites are likely to fall well outside the historical range of *P. umbrina* and as such will be defined as assisted colonizations [2].

A challenge facing conservation managers is how to identify translocation sites where species could survive under future climates. Correlative or “climate-envelope” species distribution models (SDMs) are widely used to predict the future range of species and have been applied in the selection of sites for assisted colonizations [15]. These statistical approaches capture processes implicitly; they rely on strong (often indirect or nonlinear) empirical links between species distribution records and environmental variables to make predictions, and are consequently unsuitable for species with limited occurrence data [16,17]. Notably the many rare and/or endangered species that could be candidates for assisted colonizations will often (like *P. umbrina*) have naturally restricted distributions. A further limitation is that distribution models based on correlations can produce misleading predictions in novel or non-equilibrium situations such as climate change [18,19,20]. Predicting suitable translocation sites for rare species under uncertain future climates is therefore especially challenging, but it is critical that we develop these skills expeditiously because assisted colonizations that are technically and economically feasible will be prioritized by conservation agencies if the risk of *in situ* extinction is high [5,21].

Mechanistic SDMs are a compelling alternative to the many correlative methods for predicting future habitats. Mechanistic SDMs connect ecophysiological knowledge of climatic tolerances with spatial environmental data through the application of microclimate and heat/energy budget models and can be used to define and map species’ fundamental or “thermodynamic” niche [22,23]. A major limitation of these models is the requirement for data on physiological tolerance limits and other environmentally determined thresholds, but such data is often well resolved for high profile threatened or invasive species [24,25]. Moreover, mechanistic SDMs can also simulate key components of the habitat, as in cases where temperatures of water bodies modeled as a “bucket” were used to drive thermal responses of amphibians [25,26].

In this paper we outline a mechanistic (*i.e.*, “process explicit”) framework that numerically simulates the thermal and eco-hydrological processes of a wetland and its interactions and feedbacks with the hydrothermal processes of *P. umbrina*. This complexity is necessary because the projection of the hydroperiod drives critical components of the tortoise’s heat and energy budget that in turn influence processes such as growth and reproductive provisioning. Hatchlings in particular must have a sufficiently long growing season in water to reach a critical mass before their first summer aestivation [10], and females will reabsorb their eggs after mating, or produce fewer or no eggs if their feeding opportunities are limited [27]. Hence wetland hydroperiods and water temperatures that suit the species’ requirements should be a crucial consideration when selecting translocation sites, but accounting for the complex and non-linear eco-hydrological feedbacks that occur in wetland systems experiencing a non-stationary climate regime is particularly challenging [28].

**Figure 1:**
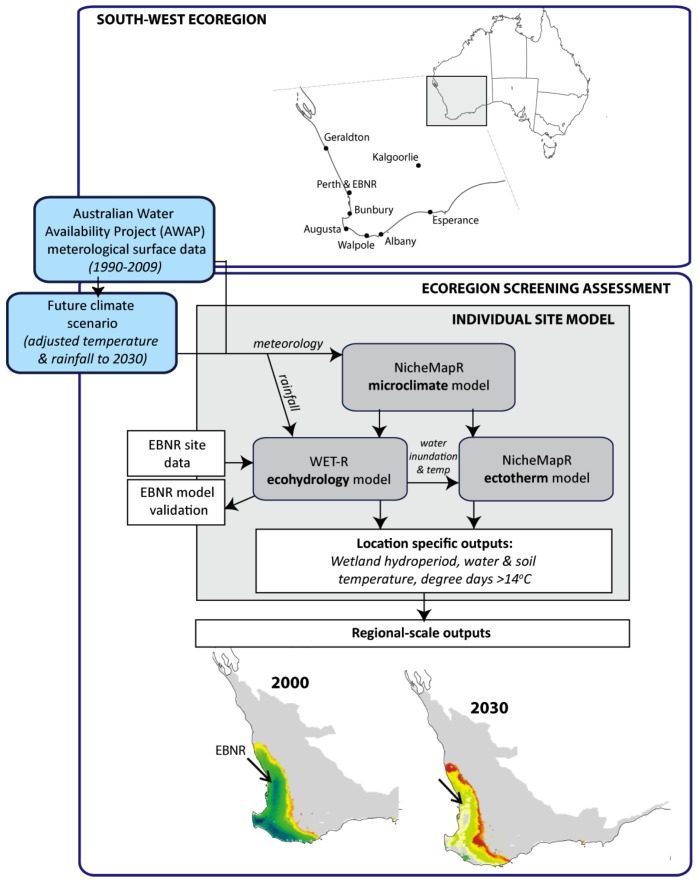
Framework coupling the eco-hydrological model WET-R to NicheMapR, where the former was validated by comparing actual hydroperiods and water temperatures at Ellen Brook Nature Reserve (EBNR) to those generated by the model. Final screening models of the Southwest Australia Ecoregion indicate relative site suitability for *P. umbrina* and are integrated using a GIS and supercomputing framework and driven using 20 years of daily climate surfaces (1990–2009) generated through the Australian Water Availability Project (AWAP [29]). Hydroperiod and water temperature outputs can be adjusted in accordance with projections of future changes in temperature and rainfall, while predictions of degree days >14 °C depend upon the behavioral routine coded in to the ectotherm model of NicheMapR.

To provide this information we employ an eco-hydrological model that is able to simulate the long-term response of a wetland’s water balance and vegetation assemblage to shifts in climate forcing and couple it to a biophysical model that simulates the heat budget of the tortoise (Figure 1). This coupling provides a sophisticated connection between the tortoise’s thermodynamic niche and the wetland water balance, as governed by climate, geomorphological and vegetation controls. We further examine three conceptual models of how tortoises regulate their body temperature during the hydroperiod to assess the activity potential of *P. umbrina* across more than 13,000 screening locations in the Southwest Australia Ecoregion. We drive our coupled models with 20 years of historical daily climate data centered on the year 2000, as well as with climate projections to 2030 to identify the parts of the ecoregion where tortoises could operate within their preferred temperature range within a suitable hydroperiod. While our model outputs are specific to *P. umbrina* and are discussed in the context of assisted colonization, the construct and approach we outline here can be generalized to any species dependent on wetland habitats. We also articulate the open questions and new understandings (both climatic, eco-hydrological and physiological) that will be required before such translocations could occur with confidence.

## 2. Results and Discussion

### 2.1. Model Performance and Assessment of Current Habitat

**Figure 2:**
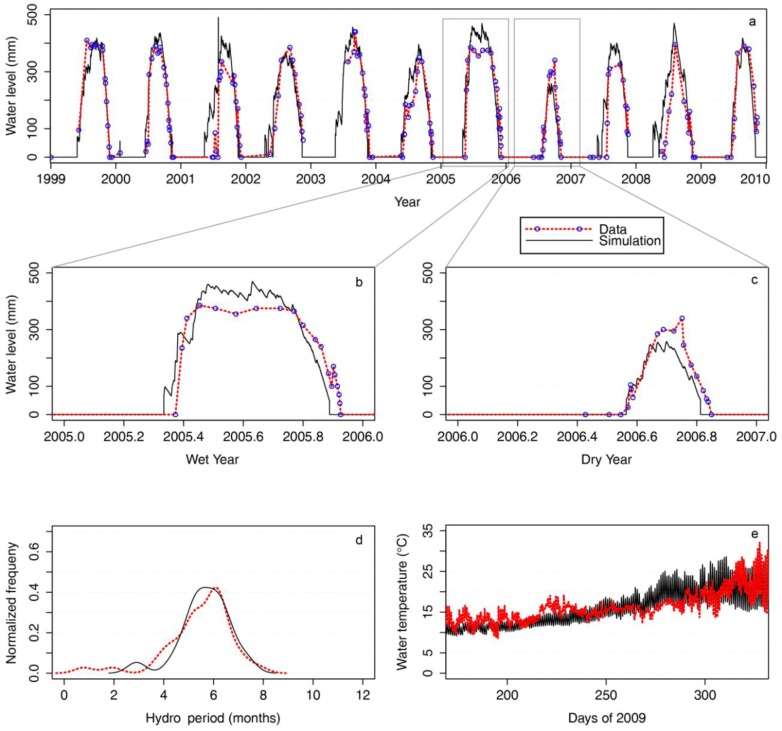
Time series of modeled (solid black line) and actual (red dotted line) hydroperiods and water temperatures at Ellen Brook Nature Reserve (EBNR). (**a**) water levels and hydroperiods of EBNR from the WET-R simulation from 1999 to 2009; (**b**) and (**c**) magnified views of water levels and hydroperiods for a wet (2005–2006) and dry (2006–2007) year; (**d**) normalized frequency of actual and simulated hydro periods (months) over 1999–2009; (**e**) actual and simulated water temperature during the 2009 hydroperiod (days 175–326).

The first step in developing our framework was to test the ability of the eco-hydrological model (WET-R—see Experimental Section) to predict hydroperiods, water depths, and water temperatures of the current habitat of *P. umbrina*. The wild population occupies two ephemeral swamps in the 80 ha Ellen Brook Nature Reserve (EBNR) and 155 ha Twin Swamps Nature Reserve (TSNR) on the Swan Coastal Plain of Western Australia [11]. We developed an eco-hydrological model for EBNR because this site currently provides the best natural habitat for *P*. *umbrina* [11]*.* The calibrated model had an Root Mean Squared Error (RMSE) of 69.9 mm between the observed and predicted hydroperiod values over the ten-year period for which we had relevant data from surface water gauging stations (1999–2009: Figure 2a–c). The frequency distribution of hydroperiod length over this period was also captured well (Figure 2d), with a peak frequency of approximately 6 months indicating the model captured the amount of inter-annual and intra-annual variability. We were only able to assess the thermal predictions of the model in 2009 (where we had continuous measures of water temperatures in the deepest part of the wetland), but seasonal trends in water temperatures and the magnitude of diel temperature fluctuations were comparable (Figure 2e), especially considering the model prediction represents a swamp average condition rather than the specific point where the sensor was recording. The ability of the model to hindcast, and therefore potentially forecast, the key microhabitat parameters at this site are clearly demonstrated.

**Figure 3:**
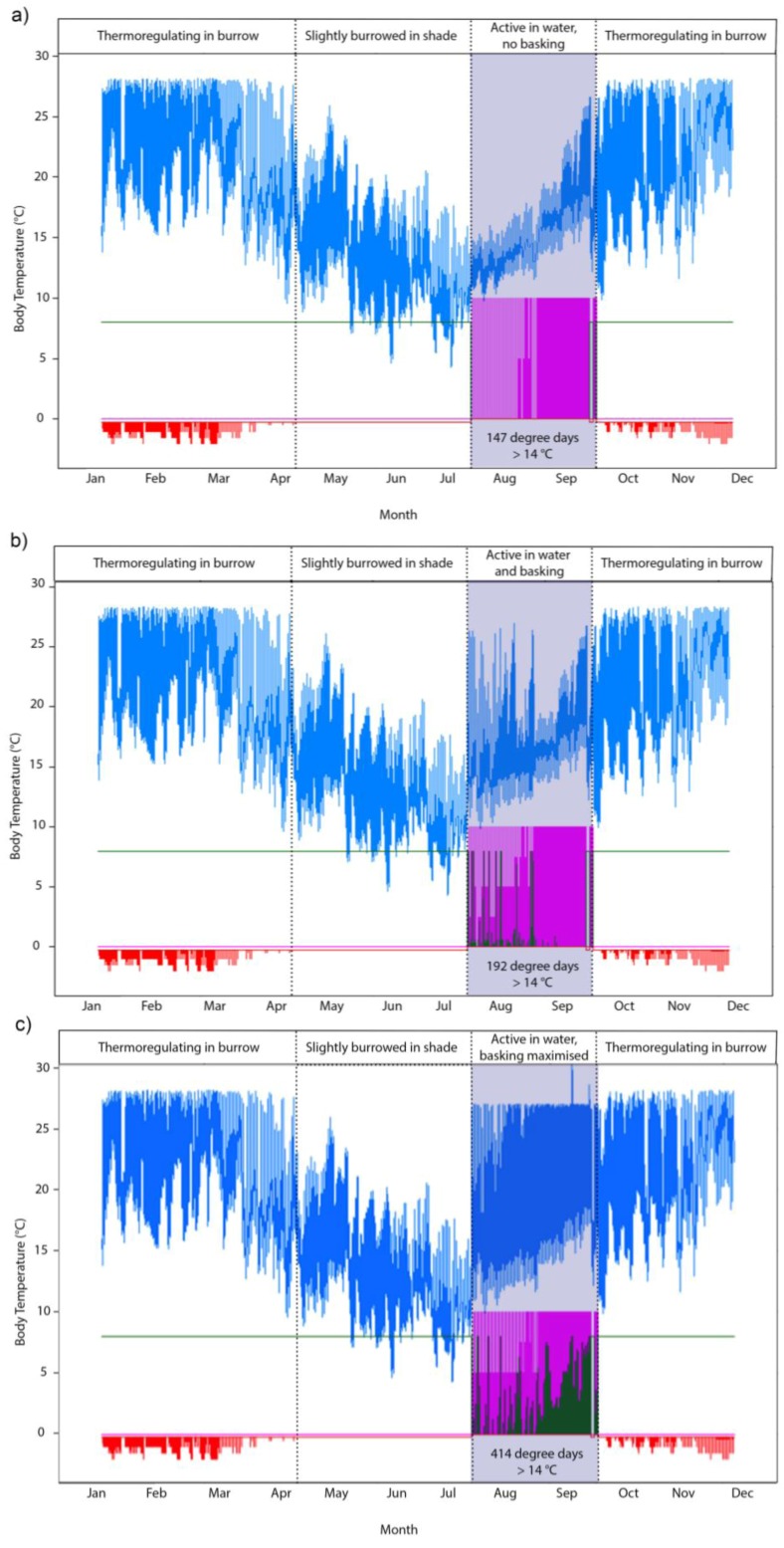
Examples of three simulations of tortoise body temperatures and activity potential at Ellen Brook Nature Reserve using AWAP climate data for 2006 when low winter rainfall severely restricted the swamp hydroperiod (grey shading). Blue lines depict the body temperature (°C) of a tortoise, pink indicates that the tortoise in is water, green indicates selected shade (10 = 100%) and the red panel shows the depth (cm) of the microclimate selected by the tortoise during the aestivation period. The behavioral routines in NicheMapR cause a tortoise to select a microhabitat that maintains its body temperature below its voluntary thermal maximum of 28 °C (Table 2). At high environmental temperatures the tortoise aestivates and thermoregulates by seeking cooler microhabitats in burrows in up to 80% shade. At lower environmental temperatures the tortoise aestivates just below the surface and ceases to thermoregulate. The tortoise then selects an aquatic environment once the wetland water depth is at least 5cm. (**a**) depicts a scenario where the simulated tortoise was prevented from basking, (**b**) demonstrates a more natural scenario where the tortoise was able to raise its body temperature above its voluntary minimum through ambient basking, while (**c**) presents a hypothetical scenario where basking is maximized (*i.e.*, simulated tortoises preferentially bask rather than swim/forage).

The projected hydroperiods over the time frame of the AWAP historical daily climate database (1990–2009) ranged from 7.8 months in 1992 to only 3.0 months in 2006 (Table 1). The consequence of the very short hydroperiod in 2006 (a year of record low winter rainfall in southern Australia [30]) for the activity potential of tortoises becomes evident when the eco-hydrological model is coupled with the biophysical model. Figure 3 demonstrates for the year 2006 how variation between three behavioral subroutines within the ectotherm model in NicheMapR (Figure 1) drove our predictions of a tortoises’ body temperature and “activity potential”—the degree days >14 °C (expressed as the number of days above the threshold temperature of 14 °C, multiplied by the number of degrees above that threshold during the hydroperiod). The body temperature of the tortoise tracks an identical pattern during the terrestrial phase of the annual lifecycle (October–July) but varied during the hydroperiod because we made different assumptions about the basking behavior of *P. umbrina* (Figure 3). The activity potential is substantially greater if the tortoise prioritizes basking over swimming and foraging (scenario **c**), whereas there was only a slight difference in scenario **a** and **b**, indicating that the option to bask in this particular year would have done little to increase the potential for activity. Notably, 2006 was also marked by an especially cold winter in South Western Australia [29].

**Figure 4:**
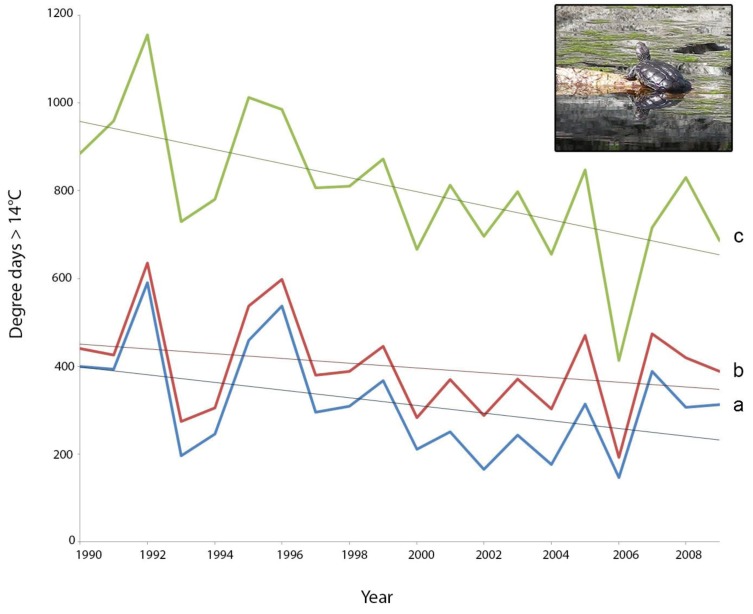
Sensitivity analysis showing how the structure of the behavioral subroutine in NicheMapR influences the degree day predictions over 20 years at Ellen Brook Nature Reserve. The blue line indicates no basking (scenario **a** in Figure 3), the red line indicates an option to bask when water temperatures fall below 14 °C (scenario **b** in Figure 3), and the green line indicates that tortoises always seeks the warmest thermal environment above a threshold of 14 °C, which would usually be out of the water (scenario **c** in Figure 3; e.g., inset photograph of a basking Western Swamp Tortoise by GK).

When comparing activity potential across the 20 years with historical daily climate data, the anomalous year of 2006 is particularly apparent (Figure 4). In 2006, the activity potential was 41%–49% less than in the much wetter preceding year of 2005 (values depending on the behavioral scenario used) where the hydroperiod was 6.75 months (Figure 4, Table 1). Limited activity is manifest in reduced opportunities for feeding and assimilation, which can be assessed by measuring growth. For example, a study of *P. umbrina* in the mid-1960s revealed that hatchling tortoises grew to only 17.1 g in a three month hydroperiod in 1966, whereas hatchlings were able to attain masses of 60 g after a 6.5–7.0 month hydroperiod in 1964 [31]. Although we have no direct evidence that tortoises’ activity or growth was limited in 2006, once of us (GK) found an exceptionally large number of *P. umbrina* carcasses at EBNR over the 2006/2007 dry season (four females, two males and four juveniles, only two of which were clearly predated). Such large numbers of carcasses have not been found either before or after 2006, over more than 15 years of monitoring. Potentially some animals were in energy deficit over the long 2006–2007 dry period, and may have died of starvation.

**Table 1:** Summary of the hydroperiod and water temperatures predicted by WET-R for the years 1990–2009 at Ellen Brook Nature Reserve.

Year	Hydroperiod (mo.)	Average water temperature °C
1990	6.45	15.43
1991	6.08	15.17
1992	7.77	15.78
1993	5.36	14.48
1994	5.51	14.48
1995	6.74	15.17
1996	5.82	15.85
1997	5.38	14.65
1998	5.61	15.14
1999	6.03	15.08
2000	5.06	14.99
2001	6.51	14.93
2002	6.57	14.63
2003	6.17	14.80
2004	5.67	14.53
2005	6.75	14.80
2006	3.02	15.36
2007	5.09	15.10
2008	7.26	14.72
2009	4.49	14.75
**Mean**	**5.87**	**14.99**

While the values in Figure 4 are based on our coupled model rather than empirical data, they suggest that there has been a decline in the activity potential during the 20-year period 1990–2009. We tested for significance of the relationship between time (year) and the degree day metric for each of the three behavioral routines, and confirmed that a linear decline was apparent for the basking-only routine—scenario C (R^2^ = 0.35, n = 20, *p* = 0.005). This result was also robust to the exclusion of the extremely low value in 2006 (R^2^ = 0.32, n= 19, *p* = 0.01). The relationships between year and degree days for scenarios **a** and **b** were either marginal or non-significant (*p* = 0.06 and *p* = 0.22 respectively). Driving the analysis with additional climate data for 2010–2012 (2010 was also a very dry winter in south-western Australia) may make the currently marginal decline significant, but daily climate surfaces for these years are not yet available. *P. umbrina* is a long lived species [11] and certainly has the capacity for persisting in a boom/bust environment, but detection of a climatically-driven downward trend in activity potential would provide a clear motivation for assisted colonization.

### 2.2. Screening of Wetland Hydroperiods under Current and Future Climates

**Figure 5:**
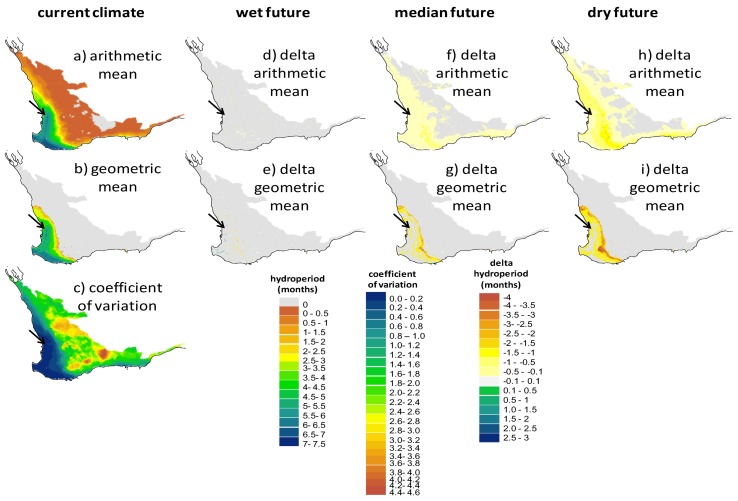
Screening maps of the Southwest Australia Ecoregion showing (**a**), the arithmetic mean, (**b**) the geometric mean and (**c**) the coefficient of variation of the hydroperiod (in months) predicted by WET-R in the period 1990–2009. Models are driven by current climate (1990–2009: AWAP data, **a**–**c**) and also indicated are the impacts on the hydroperiod of three future climates for 2030 where air temperatures increase by 0.7–1.3 °C and total annual rainfall decreases by 1%–14% (future scenario—current scenario; **d**–**i**). The black arrow indicates the location of the wild populations.

The coupled model system was applied to the Southwest Australia Ecoregion to assess how a wetland environment identical to EBNR would perform under different climatic forcing conditions. Across the ecoregion there was a large variation in mean hydroperiod lengths under the current climatic conditions, ranging from 0 to >7 months, with a sharp transition occurring along the line from Geraldton in the north, to Albany in the south (refer to Figure 1 for locations). This is exemplified most clearly in the region defined by the geometric mean (Figure 5b), based on the strong influence of rain-bearing frontal systems influencing the coastal part of southwestern corner of Australia, and the increasing potential evaporation as a function of distance inland and latitude. Beyond the Geraldton-Albany line the coefficient of variation (Figure 5c) also increased considerably, highlighting the increased level of inter-annual variability. The region on the Swan Coastal Plain and the southern coast, extending from Perth in the north to Denmark in the south, had an average hydroperiod of six months or greater, and the current EBNR site is therefore at the northern most tip of this region.

The change in hydroperiod length by 2030 varied substantially between the three future climate scenarios tested and varied from no discernible change to greater than a two month reduction. Under the wet future climate, there was no large shift with small areas improving in the southwestern tip and areas on the inland side of the six month hydroperiod contour reducing slightly (Figure 5e). Under the median future climate, there was a consistent reduction in the arithmetic mean across the ecoregion of 0.5–1 month, however the geometric mean showed a more marked change at the inland sites of up to 4 months and less significant reductions in the center of the region. Under the dry future scenario, the pattern was similar but a more extensive reduction was predicted.

### 2.3. Screening of Tortoise Activity Potential under Current and Future Climates

**Figure 6:**
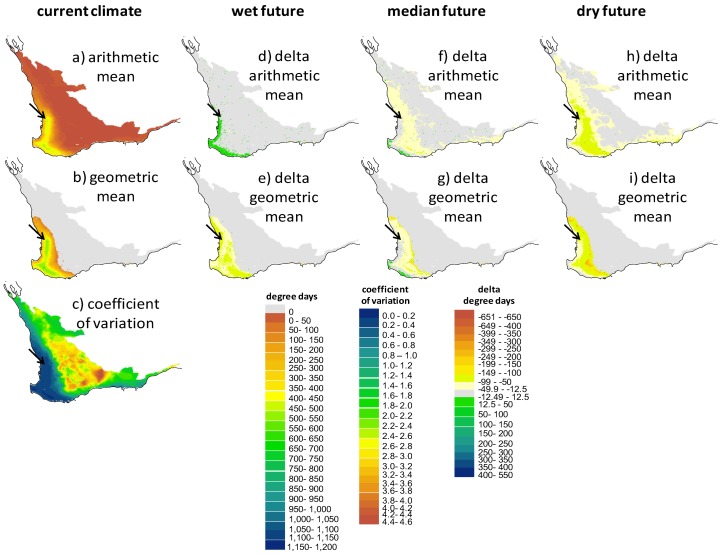
Screening maps of the output of NicheMapR coupled with WET-R showing (**a**), the arithmetic mean, (**b**) the geometric mean and (**c**) the coefficient of variation of the degree days above 14 °C, assuming the tortoises will attempt to raise body temperature by basking during the hydroperiod if water temperature was unsuitable for foraging (<14 °C). The degree day metric is proportional to the potential for feeding and assimilation*.* Models are driven by current climate (1990–2009: AWAP data, **a**–**c**) and the three future climates for 2030 outlined in Figure 4 (future scenario—current scenario; **d**–**i**).

**Figure 7:**
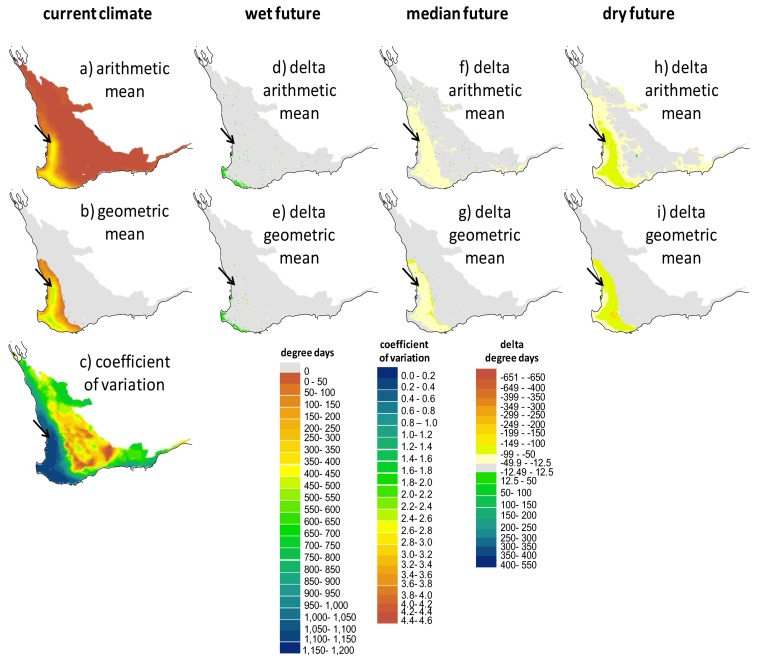
Screening maps of the output of NicheMapRcoupled with WET-R showing (**a**), the arithmetic mean, (**b**) the geometric mean and (**c**) the coefficient of variation of the degree days above 14 °C, assuming the tortoises will remain in the water during the hydroperiod unless it exceeds the voluntary thermal maximum temperature. The degree day metric is proportional to the potential for feeding and assimilation*.* Models are driven by current climate (1990–2009: AWAP data, **a**–**c**) and also indicated are the impacts of the three future climates for 2030 outlined in Figure 4 (future scenario—current scenario; **d**–**i**). The black arrow indicates the location of the wild populations.

**Figure 8:**
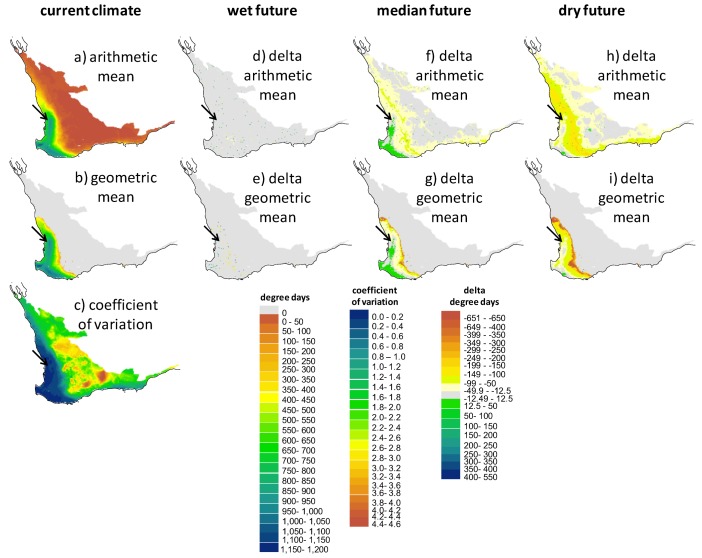
Screening maps of the output of NicheMapR coupled with WET-R showing (**a**), the arithmetic mean, (**b**) the geometric mean and (**c**) the coefficient of variation of the degree days above 14 °C, assuming the tortoises spend virtually all of the hydroperiod basking (*i.e.*, foraging bouts are only a small fraction of each simulated hour). The degree day metric is proportional to the potential for feeding and assimilation*.* Models are driven by current climate (1990–2009: AWAP data, **a**–**c**) and also indicated are the impacts of the three future climates for 2030 outlined in Figure 4 (future scenario—current scenario; **d**–**i**). The black arrow indicates the location of the wild populations.

The terrestrial and aquatic temperature predictors integrated with the behavioral routine allowed us to make spatially explicit predictions of a tortoise’s annual potential for activity (foraging and assimilating) by quantifying the extent that sites would allow tortoises to forage in water and attain their preferred body temperature range. Because the hydroperiod drives activity potential, there are obvious parallels between the maps that show the spatial variation in the hydroperiod (Figure 5) and the degree day maps in Figure 6, Figure 7, Figure 8. The key difference in these two types of screening maps is that regions that are more suitable based on thermal criteria can be distinguished. Considering the maps in the “no basking” scenarios in Figure 6 we find that for some locations, such as the coastal regions of the south west and the southern parts of the Swan Coastal Plain, the activity potential increases under the “wet future” climate, whereas there is no equivalent increase at the location of the wild populations. In this climate change scenario rainfall was reduced by only 1%, so the increase in activity is due to the projected increase in air temperature. However, under the dry future climate, the reduction of the hydroperiod length effectively offsets the increase in air temperature, with the net result being that activity potential is decreased. Under all climate change scenarios there are sites within the Southwest Australia Ecoregion where activity potential either increases relative to the present day situation (1990–2009) or decreases less than it does at the current tortoise habitat. In particular, sites in the coastal regions around Bunbury, the Scott Coastal Plain east of Augusta, and Nuyts Wilderness northwest of Walpole (see Figure 1 for locations) offer the most robust habitat (in terms of activity potential) under the three 2030 climate change scenarios we examined.

What is more notable is the effect of the assumptions made about thermoregulatory behavior (e.g., Figure 3 and Figure 4) on the degree day outputs of NicheMapR. As shown graphically in Figure 3, activity potential is enhanced when tortoises are either allowed or forced to bask (behavioral scenarios B and C). Figure 8a illustrates the much greater predicted activity potential (degree days >14 °C exceeding 1,000 in some locations) when tortoises bask continuously during the hydroperiod, and the wide band of “improved” habitat (green shading) under the median future climate scenario. Given that the behavior of continuously basking is unlikely to be realistic, the most careful attention should be paid to scenarios mapped in Figure 7, where the optional basking behavior typical of *P. umbrina* was modeled. In these cases, outcomes are similar to those described for Figure 6 (no basking) where sites near the south west coast show either no change or improved potential activity under future climates. In contrast, there was a decline in the potential for activity at the site of the current habitat at Ellen Brook Nature Reserve.

### 2.4. Implications of Screening on the Selection of Sites for the Assisted Colonization of P. umbrina

Translocations of *P. umbrina* to new habitats first began in 2000, and on average 20–40 captive-bred juveniles are introduced each year to one of two reserves about 50 km north of the known range of the species (Mogumber and Moore River Nature Reserves) or have been used to supplement the declining population at TSNR [11]. All current release sites are expected to provide increasingly marginal habitat because of decreasing winter rainfall and increasing groundwater abstraction [12]. For example, constant pumping of bore water has been necessary to maintain water levels at TSNR since 2003, and this site, which formerly provided good habitat for *P. umbrina* in the mid-1960s, now recruits very few juveniles into the population. The exceptionally low rainfall in 2006 prevented the species from breeding in all but EBNR and a drying climate was isolated as the major reason for the failure of previous recovery efforts to meet the key criterion of >50 adults in the wild [11]. Therefore the identification of translocation sites that will offer some stability and refuge under future climates will be integral for the planning of future conservation efforts, and will likely play a defining role towards *P. umbrina* recovery. 

We have shown here that it is possible to produce a species distribution model using a process-explicit rather than a statistical approach that identifies locations in terms of multiple regressions against key environmental variables. The crucial advantage, as well as the necessity of this approach for this particular case study, is that we can look at future projections with a clear understanding of at least one constraint on the tortoises’ fundamental niche. Under the three 2030 climate scenarios examined here we derive different predictions about future changes in hydroperiods and the activity potential of *P. umbrina.* The model’s predictions of the regions where suitable growth conditions occur highlights that the current population is at the northern-most margin of a large area where the climatic niche is similar, and that translocation sites north or inland of the current location are going to be unsuitable in the future under forward climate projections. 

Our model projections occur within a global biodiversity hotspot [32], where numerous species compete for conservation attention. A limitation of our climatically-driven screening model is that it does not account for a range of other typically local scale factors that will be important in selection of a translocation site for *P. umbrina*. Tools such as Multiple Criteria Analysis [33] can be used to further refine site selection, by ranking sites against criteria weighted by their importance in decision-making. Criteria relevant to translocation site selection in *P. umbrina* include soil type, vegetation composition, predator control, land tenure and anticipated land-use change, food resources, local scale hydrology and size [34] which can be assessed by liaison with stakeholders. The intersection of our spatial model outputs with sites scoring highly in relevant criteria can provide a quantified means of refinement during the final stages of selecting sites for translocations.

### 2.5. Caveats of the Existing Framework and Future Directions

This paper presents an outline or framework, and not a conclusive implementation of translocation decisions for the Western Swamp Tortoise. Refinements in the form of improvements to the climatic, eco-hydrological and physiological assumptions underlying the model must be made in order for the models to be implemented with certainty sufficient to justify a change in current practice.

The climate data used for the current data analysis is from the AWAP dataset, which interpolates daily meteorological data onto a continuous surface. In this study the daily data used at each point was further disaggregated to hourly for the microclimate and wetland models by simply assuming the daily rainfall was spread evenly over the day, however, it is well known that sub-daily patterns of rainfall distribution can vary over the ecoregion assessed here [35]. Since runoff generation in the wetland region is essentially a threshold process often governed by sub-hourly patterns of rainfall intensity and antecedent conditions, spatial and temporal variability in the nature of rainfall delivery may impact on the overall frequency of wetland filling and should be further considered as part of a local scale hydrological assessment.

Spatial changes in the temporal character of rainfall delivery may also occur under future climate scenarios. For example, an increase in dominance of the rainfall from summer thunderstorm activity versus winter frontal systems may significantly impact on the nature of runoff generation and overall hydroperiod length. In the present analysis we adopted a simple increase in temperature and decrease in rainfall based on 2030 conditions [8], which were average regional changes based on the ensemble prediction of 15 GCM’s. Alteration to the seasonal character and sub-daily intensity patterns of rainfall delivery have therefore not been considered in our assessment and may significantly impact on the timing of wetland filling, the lake water balance and length of the hydroperiod. Similarly, our approach does not consider heterogeneity in the spatial response of the region to a changing climate; meso-scale changes in land surface topography and condition may locally amplify or dampen projected shifts in rainfall and temperature. Therefore, we propose to use appropriate GCM downscaling approaches [36] to account for these changes. Further, we propose also to account for uncertainty in GCM predictions by running a Montecarlo simulation of the combined effects of declining rainfall and increasing air temperatures, sampled from assumed normal distributions parameterized from the percentiles of the GCM predictions.

A key assumption of our screening is that the characteristics of the Ellen Brook wetland are transferrable throughout the southwest of Western Australia. Here we have assumed identical features of the wetland hydrological processes; that is, the claypan nature of the wetland with limited surface water and groundwater, its soil and vegetation properties, as well as the wetland depth-area-volume relationship. This is a logical approach as it is likely to be the most pessimistic case. Calibration of our model has showed that hydroperiod is very sensitive to soil hydraulic properties as well as variations in the wetland geometry, and Coletti *et al*. [28,37] has also demonstrated the sensitivity of different morphologies, soil and vegetation properties to wetland inundation under different climatic conditions, to which the reader is also referred. Local variables may create better or worse microclimatic and wetland conditions and should be considered in addition to screening based purely on climatic differences—application of the model to specific wetlands would require site specific data and individual calibration. Alternatively, it may be possible to optimize the claypan wetland geometry to maximize the performance of *P. umbrina* in each climate. Such an assessment may help limit the number of potential release sites or provide a design for an engineered wetland could that be a viable option for assisted colonization if natural sites are limited.

Our physiological analysis centers on the heat budget and how it interacts with the hydroperiod and water temperatures to permit aquatic activity at suitable body temperatures. While our predictions for degree days of foraging time provide key insights into the relative suitability of different parts of southwestern Australia as potential habitat for *P. umbrina*, they do not provide a means to identify key thresholds of suitability. The latter will require the development and integration of energy and water budgets to quantify the consequences of different hydroperiods for growth and reproduction potential as well as the consequences of the dry periods for loss of body condition in terms of energy/nutrient and water stores. In this context, Dynamic Energy Budget (DEB) theory [38], a formal theory and model for metabolism, will play a key role in integrating heat, water and mass budgets across the entire life cycle. It will provide a means to capture the effect of different climatic histories on future growth and reproduction and to capture phenological constraints in the context of the full life cycle [23,39]. This in turn will allow us to predict how recruitment and survival respond to annual variation in temperature and rainfall, especially in terms of the energy available to females for egg production and the potential for growth in the hatchlings’ first season and its influence on survival through their first summer. Such predictions can then act as input to population viability models. The merger of DEB with NicheMapR has already been demonstrated for an ectotherm model [23] and, whilst beyond the scope of this paper, the integration of DEB into our existing model is a logical next step for improving the predictions generated here.

The requirements of the terrestrial phase of the annual lifecycle of *P. umbrina* have largely been ignored here for the purposes of explaining our framework for coupling eco-hydrological and ecoenergetic models. Nonetheless, understanding the constraints of the terrestrial environment will be as critical as understanding the spatial variation in activity potential. Tortoises spend around half of the year (more in dry years) in terrestrial environments that fringe the wetlands. Eggs develop in underground nest chambers [40] and juvenile and adult tortoises seek out cool microclimates during this period and aestivate under leaf litter or in existing burrows or crevices. During this period eggs and tortoises are vulnerable to energy depletion, hyperthermia and desiccation. Both NicheMapR and WET-R have existing capacity to predict soil temperature and water potential, and a database of soil properties across Australia at 5 km resolution offers further avenues to make such predictions spatially explicit. Modeling the heat and energy balance of tortoises when in terrestrial habitat, and integration of DEB theory in NicheMapR, will ultimately allow us to model processes such as allocation of energy to the reproductive buffer (eggs) during aestivation.

## 3. Experimental Section

### 3.1. Wetland Water Balance and Thermodynamic Model: WET-R

The wetland model applied here is based on Coletti *et al*. [37] which divides the wetland into different zones of hydrological function and simulates partitioning of water between the zones as mediated by climate, soil and vegetation controls. As a result, the model predicts the changes in the extent of surface water inundation, soil moisture and vegetation, and captures the various eco-hydrological feedbacks that mediate wetland response to changes in rainfall delivery and air temperature [28]. In this analysis, the above model was adapted to operate on a sub-daily (hourly) time-step and to simulate wetland thermodynamics. 

Water temperature is computed based on a heat budget considering solar heating, longwave radiation and standard bulk aerodynamic flux parameterizations for sensible and latent heat. The budget is driven by the prevailing meteorological conditions provided by the microclimate model of NicheMapR, and the heat balance is calculated as:

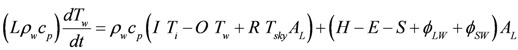
(1)
where *T_w_* is the lake temperature, *T_i_* is the inflow temperature, *T_sky_* is the air temperature (all in °K), *p_w_* is the density of water (kg/m^3^), *c_p_* is the specific heat capacity of water (J/kg/K), *L* is the lake volume (m^3^) and *I*, *O* and *(A_L_R)* are the inflow, outflow and rainfall volume fluxes respectively (m^3^/d), where *A_L_* is the lake surface area (m^2^) used to multiply the rainfall depth (m/d). *H*, *E* and *S* are heat fluxes (W/m^2^) for sensible heat, evaporation and soil heat conduction, respectively, calculated according to:

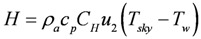
(2)

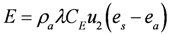
(3)

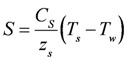
(4)
where *C_H_*, and *C_E_* are bulk transfer coefficients for sensible and latent heat respectively (-), *λ* is the latent heat of vaporization (J/kg), *ρ_α_* is the density of air (kg/m^3^), and *C_S_* is the diffusivity of heat into the soil below the water (W/m/K), and z_s_ is the active soil depth over which heat diffusion occurs. *ϕ_LW_* is the net longwave radiation (W/m^2^) and *ϕ_SW_* is the solar insolation (W/m^2^), calculated from:

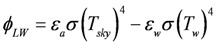
(5)

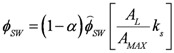
(6)
where *ε* is the emmisivity of air or water, *σ* is the Stefan-Boltzman constant, *α* is the shortwave radiation albedo, 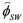 is the incident solar radiation above the vegetation (W/m^2^). The last term of Equation 6 accounts for the effect of wetland vegetation shading on the incident solar intensity, with *k_s_* defined as a constant to increase the shading effect, and *A_MAX_* is the maximum area of inundation extent (m^2^).

### 3.2. WET-R Setup and Calibration

The wetland morphology, soil, vegetation and initial conditions were based on application of the model to the wetland system at EBNR. A digital elevation model of the area at one meter resolution was obtained from the Western Australian Department of Environment and Conservation (DEC) and was used to derive the area-depth-volume curve for the model. The hydrological and vegetation parameters were applied as in Coletti *et al.* [37], where the model was similarly applied across the range of environments representative of the region. The wetland has previously been identified as a claypan system, with no significant inflows from surface waters or ground waters, but historically surface water and groundwater have likely contributed [41]. Rather, it is considered to be a bowl, which fills seasonally from direct rainfall and due to the low permeability of the sediments, it drains slowly to groundwater and more rapidly due to evaporation. Weirs installed at a height of 450 mm above the base of the wetland limited the depth of inundation. These factors greatly simplified the WET-R model and reduced the number of uncertain parameters.

Water level observations taken at EBNR for the years 1999–2009 were used to calibrate the water balance sub-model. Observations were derived from visual inspection of up to eight level gauges installed in the center of deeper pools throughout the wetland, and were made at approximately monthly intervals. Calibration of WET-R involved adjustment of three hydraulic parameters and five thermal parameters. The three hydraulic parameters calibrated included the saturated hydraulic conductivity of the lake sediments (*h_c_*, 0.1 mm/h), the adjacent sandy sediments controlling internal water redistribution (*h_cu_*, 0.11 mm/h) and the exponent on infiltration rate relation to saturation (*k_i_*, 3.0). A simulated annealing algorithm was used to obtain the best-fit of these hydraulic parameters by reducing the root mean square error (RMSE) between observed and modeled water levels [42,43].

Water temperature was been logged every 15 minutes at EBNR since early 2009 using an automated weather station (Unidata 6540V) with a linear temperature probe positioned in the deepest part of the wetland. For this period (2009) we also ran WET-R using the locally measured meteorological data generated by the on-site weather station (rather than the AWAP climate surface) to provide the most accurate assessment of the performance of the model in predicting wetland water temperatures. Assumed values for *C_S_*, *C_H_*, *C_E_*, ε_a_, *k_s_* were 0.5, 0.0013, 0.0013, 0.99 and 0.25, respectively.

### 3.3. Modeling the Heat Budget and Activity Constraints of P. umbrina

Available physiological and behavioral data for *P. umbrina* (Table 2) was used as input for a biophysical model within the software package NicheMapR (an R version of the package Niche Mapper™ [44]) which solves heat and mass budgets under different microclimatic scenarios for given behavioral strategies [23,44,45,46]. The problem of defining the microclimates available for a particular species can be tackled by combining weather station data with microclimate models. For the *P. umbrina* case study here we can drive the microclimate model (described in [45]) of NicheMapR with historical continent-wide 0.05° grids of daily minimum and maximum temperature, vapor pressure, rainfall and daily solar radiation available through the Australian Water Availability Project (AWAP, [29]). The microclimate model calculates the clear sky solar radiation from first principles, and we can account for cloud cover effects on solar (and infrared) radiation exposure through the AWAP daily solar estimates (*i.e.*, observed daily solar over calculated clear sky solar). In the absence of interpolated daily wind speed data we use gridded long-term average wind speed obtained from ANUCLIM [47]. The microclimate model runs for two extremes of shading. We used a fixed value of 80% shade as the maximum value and characterized the minimum available shade using daily interpolations of monthly long-term average (1995–2008) values of the satellite derived Fraction of Available Photosynthetically Active Radiation (FAPAR) [48,49]. The minimum shade values derived from FAPAR influenced the soil heat budget but we assumed that basking tortoises could find patches of full sun. In sum, these microclimate calculations provide realistic hourly estimates of the microclimatic conditions above and below ground available to *P. umbrina* over a 20-year period from 1990 to 2009. However, because the microclimate model in NicheMapR does not explicitly consider microclimates available in a wetland setting, we derive predictions of water availability and temperature from the eco-hydrological wetland model (WET-R—outlined above). Further environmental information gained from this approach includes the wetland hydroperiod, water temperature, and feedbacks of the wetland on soil temperature, soil moisture and vegetation changes.

**Table 2:** Parameters for the biophysical model of *P. umbrina* that were used to predict the thermodynamic niche.

Parameter	Value	Source
*Morphological Traits*		
Insulative fat layer thickness	0	Default value
Thermal conductivity of flesh	0.5 W/mC	Default value
Specific heat of flesh	4,185 J/(kg-K)	Default value
Density of flesh	1,000 kg/m3	Default value
Maximum solar absorptivity	0.85%	Default value
Minimum solar absorptivity	0.85%	Default value
Emissivity of animal	1	Default value
Reflectance of animal	0.9	Assumed
Proportion of body surface area in water while basking	30%	Assumed
Configuration factor to sky	0.4	Default value
Configuration factor to substrate	0.4	Default value
*Physiological Traits*		
Voluntary thermal maximum (upper body temperature for foraging, leaving pond, seeking deeper burrows)	28 °C	[31,50,51,52]
Voluntary thermal minimum (lower body temperature for foraging)	14 °C	[50,52]
Temperature difference between expired and inspired air	0.1	Default
Proportion of surface area acting like a free water surface	1%	Default
*Behavioral Traits*		
Daily activity	Diurnal *	[51], this study
Retreat underground in 80% shade	Yes—max depth of 1 m	GK unpublished
Shade seeking	Yes—0 to 80%	[53]
*Allometric Respiration*		
Typical mass of an animal	6 g (hatchling)	[11]
Metabolic Rate	Q = 0.013 M^0.8^10^(0.038*Tb)^	[54]

* *P. umbrina* was confirmed to be diurnal by overnight video surveillance of 16 individuals at Perth Zoo for a total of 14 surveillance nights during late spring [55].

Outputs from both the microclimate model and wetland model then drive a steady-state biophysical model that solves the heat and mass balance for *P. umbrina* based on morphological, physiological and behavioral traits entered by the user (Table 2). In this study we calculated the heat budget only. The behavioral subroutine within NicheMapR was configured for *P. umbrina* by including thermal thresholds for basking and swimming (where the latter would allow foraging). We assumed that a tortoise would enter the wetland and begin activity if water depth exceeded a threshold of 0.05 m. Because heat exchange under water is dominated by convection, aquatic organisms such as *P. umbrina* will assume the temperature of the water when feeding or basking aquatically, but can elevate their body temperature by basking above the water surface or on land. If water temperatures are too low, tortoises can remain within their active temperature range by seeking out basking microclimates to raise their body temperature above the voluntary thermal minimum (14 °C; Table 2). We therefore implemented this behavior as a subroutine in the model, and further assumed that, in the absence of a suitable basking microclimate, tortoises would remain (inactive) in the water until the next hourly time-step, when they could surface to seek a basking microclimate once more. However, we also appraised two other less likely scenarios. The second scenario assumed tortoises would not bask but remained in the water at all times. The third scenario was that tortoises basked 100% of the time on the assumption that only very short bouts of foraging in the water were needed to fill the gut and that the rest of the time was spent basking to maximize assimilation rates. These additional behavioral scenarios allowed us to assess the sensitivity of our assumptions regarding basking behavior on our predictions of the thermodynamic niche.

When the water temperatures predicted by WET-R caused tortoises to exceed their voluntary thermal maximum of 28 °C, they could seek shade on land until the next hourly time-step, at which point they could return to the water if it had cooled sufficiently to reduce body temperatures to within the voluntary thermal range. Once either the hydroperiod ended or water temperatures permanently exceeded 28 °C, tortoises were terrestrial and entered aestivation, while continuing to defend an upper preferred body temperature by seeking cooler microclimates up to one meter underground in shaded burrows. We set a shade value of 80% to reflect the observation that the aestivation sites of *P. umbrina* are frequently beneath small trees or bushes [56].

### 3.4. Regional Screening for Suitable Translocation Sites

The coupled model was initially applied across the Southwest Australia Ecoregion for the period 1990–2009 using daily climate data from AWAP. These daily data were disaggregated to an hourly time step by assuming air temperature and wind speed peaked one hour after local solar noon and reached a minimum at dawn (vapor pressure and cloud were assumed to be constant through the day but to vary between days). Rainfall for a given day was spread evenly across 24 h. The simulations were run at 5 km resolution representing 13,169 locations and performed on a supercomputer (Victorian Life Sciences Computation Initiative) that allowed up to 200 sites to be processed simultaneously such that a 20-year run across the whole ecoregion took approximately 1 h.

Hourly predictions of water level and temperature from the wetland model were passed to the NicheMapR ectotherm model at each location in the screening assessment. The NicheMapR system then computed, on an hourly time step, the degree days that were within the hydroperiod and above the threshold activity body temperature of 14 °C (with an upper cap at 28 °C) during daylight hours. These were summarized per year of simulation and we then calculated the arithmetic and geometric means of hydroperiod and degree days across years as well as the coefficient of variation of the arithmetic mean. 

To compare how the spatial extent of the activity potential varied under future climate conditions we ran the above analysis for the year 2030 by adjusting the AWAP data with projected future climates based on 16 Global Climate models each driven by one of three future greenhouse gas emission levels [8]. The three scenarios were a “Wet future” climate (0.7 °C air temperature increase, −1% rainfall decrease), a “Median future” climate: (1 °C air temperature increase, −7% rainfall decrease) and a “Dry future” climate: (1.3 °C air temperature increase, −14% rainfall decrease). Daily air temperature data from the AWAP database were simply increased by the appropriate increment before running the microclimate model, and AWAP daily rainfall data were reduced by the appropriate amount. We then computed the differential between the current and future conditions for each metric.

## 4. Conclusions

Planning for assisted colonization demands rigorous methodologies for selecting release sites, with risk assessment being foremost among a range of potential considerations [57]. Despite the fact that assisted colonization will be most often motivated by climate change, little effort has been expended in developing methodologies to forecast the future climatic suitability of a release site. Correlative SDMs have limited potential for identifying future habitats for range-restricted species, but the framework that we have demonstrated here offers a methodology for guiding the assisted colonization of any species dependent upon ephemeral wetlands. Further, our eco-hydrological model could be simplified for organisms inhabiting perennial wetlands, and our eco-energetic model can be driven by parameters for analogous species’ if the required physiological data are unavailable, provided the model outputs can be tested against a known location and population.

In the *P. umbrina* case study developed here we have located regions of suitable habitat under a range of likely future climates. Ultimately our methodology will be expanded to explicitly include the energy/mass budget of the tortoises, and local scale hydrology, to allow quantitative statements about growth, development, starvation risk and reproduction. We will also consider not only longer term climate changes as predicted by global climate models, but also the natural climate variability across a range of temporal and spatial scales relevant to the prediction of wetland hydroperiods. Such approaches can be used to determine how sensitive the suitability of a particular translocation site will be to future predictions of climate changes.
